# Smoking is not linked to the development of anti-peptidylarginine deiminase 4 autoantibodies in rheumatoid arthritis

**DOI:** 10.1186/s13075-018-1533-z

**Published:** 2018-03-23

**Authors:** Laura C. Cappelli, Maximilian F. Konig, Allan C. Gelber, Clifton O. Bingham, Erika Darrah

**Affiliations:** 1 Division of Rheumatology, The Johns Hopkins School of Medicine, Baltimore, MD 21224 USA; 2 Department of Medicine, Massachusetts General Hospital, Harvard Medical School, Boston, MA 02114 USA

**Keywords:** Rheumatoid arthritis, Smoking, PAD enzymes, Shared epitope, CCP

## Abstract

**Background:**

Defining environmental factors responsible for development of autoimmunity in rheumatoid arthritis (RA) is critical for understanding mechanisms of disease initiation and propagation. Notably, a history of cigarette smoking has been implicated in the genesis of RA and is associated with worse disease outcomes. Antibodies to peptidylarginine deiminase 4 (PAD4) are also associated with more severe RA. A subset of patients who have PAD4 autoantibodies that cross-react with PAD3 (anti-PAD3/4) are at the highest risk for interstitial lung disease, and this risk is augmented by a history of cigarette smoking. It is unclear, however, if smoking is etiologically linked to the development of anti-PAD4 antibodies.

**Methods:**

Patients were included in this study if they had physician-diagnosed RA as well as DNA, serum, and a date-matched clinical assessment (*n* = 274). Anti-PAD4 and anti-CCP antibodies were measured by immunoprecipitation and ELISA, respectively; shared epitope (SE) status was determined by HLA-DRβ1 genotyping. Logistic regression analysis was used to evaluate associations of smoking with PAD4 antibodies, with adjustment for relevant demographic and clinical features. Stratified analyses by disease duration and shared epitope status were also performed.

**Results:**

Anti-PAD4 antibodies were present in 25% of RA patients, with 50% of these individuals having anti-PAD3/4 cross-reactive antibodies. Anti-PAD4 antibodies were significantly associated with a longer disease duration, SE alleles, and anti-CCP antibodies. Importantly, there were no significant differences in smoking history between anti-PAD4 positive and negative groups in univariate analyses, stratified analyses, or multivariable models. However, an inverse relationship between smoking and anti-PAD4 antibodies was suggested by a lower prevalence of current smokers among patients with anti-PAD3/4 antibodies compared to antibody negative individuals (*p* = 0.04). Further, the lowest levels of anti-PAD4 antibodies were observed in current smokers (*p* = 0.14), and a significant association of SE and anti-PAD4 antibodies was only present among never smokers (*p* = 0.01).

**Conclusions:**

Smoking history was not associated with anti-PAD4 antibodies in patients with RA. The finding that anti-PAD4 antibodies were not associated with smoking suggests that other environmental factors may contribute to the development of autoimmunity to PAD4 in these patients.

## Background

Identifying the environmental triggers of autoimmune disease has been a focus of research for many decades. In rheumatoid arthritis (RA), a prototypic systemic autoimmune disease, several environmental exposures have been posited to contribute to disease pathogenesis including cigarette smoking, microbial infection, and microbiome alteration [1]. In predisposed individuals, these environmental stimuli have been proposed to induce inflammation at extra-articular sites such as the lung and gingiva, respectively, breaking immune tolerance to self-antigens ultimately targeted in RA [2]. Notably, anti-citrullinated protein antibodies (ACPAs), hallmark serological findings in patients with RA, have been found in the lungs of at-risk individuals prior to the onset of arthritis and are enriched in gingival crevicular fluid from patients with periodontitis [3, 4].

Smoking is the most widely studied environmental risk factor linked to RA development and prognosis [5, 6]. One way that smoking is potentially linked to RA pathogenesis is through its association with the development of ACPAs [7–9], detected by the anti-cyclic citrullinated peptide (CCP) clinical assay. In some studies, the association of anti-CCP antibodies and smoking was most pronounced in patients who harbored one or more MHC class II shared epitope (SE) allele associated with RA development [10–12]. Smoking has also been associated with more severe disease manifestations in patients with RA, most notably increased risk of interstitial lung disease (ILD). Patients with SE alleles [13] and those seropositive for RF or anti-CCP antibodies were more likely to have ILD if they smoked [14]. In addition, several studies have demonstrated that smoking correlates with higher RA disease activity and higher levels of inflammatory cytokines [15, 16].

The citrullinated protein targets of ACPA are generated by the peptidylarginine deiminase (PAD) enzyme family. In humans, there are five PAD isoenzymes, PAD 1, 2, 3, 4, and 6. PAD2 and PAD4 have been most strongly implicated in RA pathogenesis due to their ability to generate the citrullinated autoantigens targeted by ACPAs, and their enrichment in the synovia of patients with RA [17, 18]. Increased PAD enzyme expression and accumulation of citrullinated proteins has also been observed in the lungs of chronic smokers in the absence of RA, suggesting increased PAD enzyme activity as a potential pathophysiological link between smoking and ACPA development. Importantly, antibodies to the PAD4 enzyme are found in approximately 35% of patients with RA and are associated with anti-CCP, disease duration and erosive joint disease in several cohort studies [19–21]. Anti-PAD4 antibodies were found less frequently in early RA cohorts with 1.4% of at-risk individuals, 18% of patients in the pre-clinical phase, and 21% of patients with disease duration less than two years being seropositive [20]. This suggests a model whereby anti-PAD4 antibodies are not dominant initiators of disease, but rather are important amplifiers of disease severity in a subset of patients with RA. This is supported by the recent discovery of a subgroup of patients who harbor anti-PAD4 antibodies that cross-react with the related isoenzyme PAD3 (designated as anti-PAD3/4 antibodies) [22, 23]. Patients with anti-PAD3/4 antibodies had the longest disease duration and most severe and progressive erosive joint disease compared to antibody-negative groups [23]. Furthermore, anti-PAD3/4 antibodies had the unique ability to activate the PAD4 enzyme *in vitro*, enhancing the generation of citrullinated proteins. In a follow-up study, these autoantibodies were found to be associated with the presence of ILD, a risk that was augmented by a history of smoking [24].

Despite the known independent associations of anti-PAD4 autoantibodies and smoking with RA disease severity, ILD, and ACPAs, it is unknown if cigarette smoking is etiologically linked to the development of anti-PAD4 antibodies. Identification of environmental stimuli that drive the generation of anti-PAD4 antibodies is important to define mechanisms that promote disease evolution. Further, clinical intervention directed at modifiable risk factors may prevent the development of these potentially pathogenic autoantibodies. Therefore, this study was designed to systematically test the hypothesis that smoking is an important environmental trigger for the development of anti-PAD4 antibodies in patients with RA.

## Methods

### Study Participants

Patients were drawn from a RA cohort followed at the Johns Hopkins Arthritis Center (IRB # 00040493) who had physician-diagnosed RA, were 18 years of age or older, had a DNA sample available, and had a serum sample available from the same day as a clinical assessment (*n* = 274).

Patient and physician data were evaluated from the same date as available serum. Patient-reported data including global disease activity visual analog scale (VAS) and smoking history was collected by questionnaire. Smoking history was categorized as current, former, or never by patient report. The composite clinical disease activity index (CDAI) was available on all patients. Data about radiographic erosions was collected from chart review; erosive disease was designated in a binary fashion based on the presence or absence of any bone erosions on radiograph review or radiologist report.

### Anti-PAD antibody assays

Immunoprecipitation assays were used to test for anti-PAD4 and anti-PAD3/4 antibody positivity, as previously described [23, 25]. Briefly, S^35^-labeleld PAD4 protein was generated by *in vitro* transcription and translation (Promega) and was incubated with patient sera for 1 h at 4 °C. Antibodies were then isolated by protein A-agarose beads (Pierce) and immunoprecipitated PAD4 was visualized by radiography. Anti-PAD4 positive samples were evaluated for antibody cross-reactivity to PAD3 using a similar protocol by incubating sera with S^35^-labeleld *in vitro* transcribed and translated PAD3. The amount of immunoprecipitated PAD3 or PAD4 was quantified by densitometry and normalized to a known anti-PAD3/4 antibody positive serum to calculate anti-PAD antibody arbitrary units.

### Anti-cyclic citrullinated peptide (CCP) antibody assays

Anti-CCP antibodies were measured in sera using the QUANTA Lite® CCP3 IgG ELISA (iNova Diagnostics). Measurements were made using sera from the same date as anti-PAD antibody testing, and positivity was determined based on manufacturer’s instructions (negative < 20, weak positive = 20–39, moderate positive = 40–59, and strong positive ≥60).

### Evaluation for Shared Epitope alleles

High-resolution HLA-DRβ1 genotyping was performed on DNA from each patient by next generation sequencing at the Johns Hopkins Immunogenetics Laboratory. Briefly, HLA-typing was performed with the TruSight HLA Sequencing Panel (illumina®, San Diego, CA). PCR primers were used to generate long range amplicons. Post PCR products were enzymatically cleaved and each fragment was end labeled. Automated paired end sequencing by synthesis was performed on the MiSeq System. HLA types were assigned using the Assign TruSight HLA Analysis software. HLA-DRβ1*0101, 0102, 0401, 0404, 0405, 0408, 1001, 1402 were designated as SE alleles [24].

### Statistical analysis

Summary statistics were calculated for the three groups of patients: anti-PAD4 antibody negative, anti-PAD4 mono-reactive antibody positive, and anti-PAD3/4 cross-reactive antibody positive. Wilcoxon rank sum tests were used to evaluate differences in continuous variables between groups; chi-square tests were used to evaluate the categorical variables. Levels of anti-PAD-4 antibodies were compared between smoking status groups for those positive for anti-PAD4 antibodies graphically with box plots and with one way ANOVA. Logistic regression was used to determine the association between demographic or clinical variables and PAD4 antibody serologic status in univariate and multivariate models, yielding odds ratios and related confidence intervals. Stratified analyses were performed to evaluate for differing associations between smoking and PAD4 antibodies in subgroups, defined by gender, disease duration and shared epitope status. The relationship between shared epitope and anti-PAD4 antibodies was also investigated in stratified analyses according to smoking status and disease duration.

## Results

Among 274 patients in this cohort of established RA, 68 (24.8%) had anti-PAD4 antibodies. Of these, 34 (50%) had anti-PAD4 autoantibodies that cross-reacted with PAD3, for an overall frequency of 12.4%. Demographic and clinical features were evaluated in patients without PAD4 antibodies, those with anti-PAD4 mono-reactive antibodies, and those with anti-PAD3/4 cross-reactive antibodies (Table 1). Pair-wise comparisons yielded no significant differences in sex, race, or age distribution between the three groups. On average, patients were in their 50s, were majority Caucasian, and more likely to be female than male.

**Table 1:** Demographic and clinical features of patients with RA according to Anti-PAD antibody status

	PAD negative (P0) (*N* = 206)	PAD4 Ab only (P4) (*N* = 34)	PAD3/4 Ab (XR) (*N* = 34)	*p*-value P0 vs P4	*p*-value P0 vs XR	*p*-value P4 vs XR
Female sex- N (%)	163 (79%)	27 (79%)	28 (82%)	0.99	0.66	0.76
Race- N (%)				0.99	0.91	0.95
Caucasian	165 (80%)	28 (82.4%)	27 (79%)			
African American	27 (13%)	4 (11.8%)	4 (12%)			
Asian/Other	14 (7%)	2 (6%)	3 (9%)			
Age- median (IQR)	57 (49–66)	55 (47–68)	51.5 (46–64)	0.70	0.14	0.33
Disease duration (yrs) - median (IQR)	6 (2–12)	15.5 (8–27)	15.5 (7–26)	< 0.01	< 0.01	0.77
Ever smoking- N (%)	95 (47%)	16 (47%)	12 (35%)	0.96	0.22	0.32
Current smoking- N (%)	24 (12%)	3 (8.8%)	0 (0%)	0.62	0.04	0.08
CCP positive- N (%)	116 (57%)	30 (88.2%)	30 (88.2%)	< 0.01	< 0.01	1.0
Shared Epitope- N (%)	112 (54%)	28 (82.4%)	22 (64.7%)	< 0.01	0.26	0.10
Erosions- N (%) *N* = 268	88 (43%)	17 (54.8%)	22 (65%)	0.24	0.02	0.42
CDAI- median (IQR)	7.9 (3–16)	7 (2.8–11.5)	3.7 (1.5–9.5)	0.37	0.04	0.21
On a DMARD- N (%)	184 (90%)	28 (82%)	28 (82%)	0.21	0.20	1.0
On methotrexate- N (%)	127 (63%)	20 (59%)	24 (71%)	0.73	0.33	0.31
On a biologic- N (%)	89 (44%)	17 (50%)	16 (47%)	0.49	0.71	0.81

The percentage of patients with anti-CCP antibodies and median disease duration were identical among patients who were positive for anti-PAD4 mono-reactive antibodies and those with PAD3/4 cross-reactive antibodies. Patients in these groups were more likely to be anti-CCP positive and had a median disease duration 9.5-years longer than those without PAD antibodies (*p* <  0.01 for both). Patients with PAD4 mono-reactive antibodies were also significantly more likely to have one or more SE allele than those without PAD antibodies (p <  0.01). There was a higher prevalence of erosive disease in patients with anti-PAD3/4 antibodies (*p* = 0.02), but this group had a 53% lower median clinical disease activity index (CDAI) at the time of clinical examination, when compared to anti-PAD4 negative individuals (*p* = 0.04). Interestingly, there were significantly fewer current smokers in the anti-PAD3/4 positive group compared to those without PAD4 antibodies (0% vs. 12% respectively, *p* = 0.04).

**Fig. 1:**
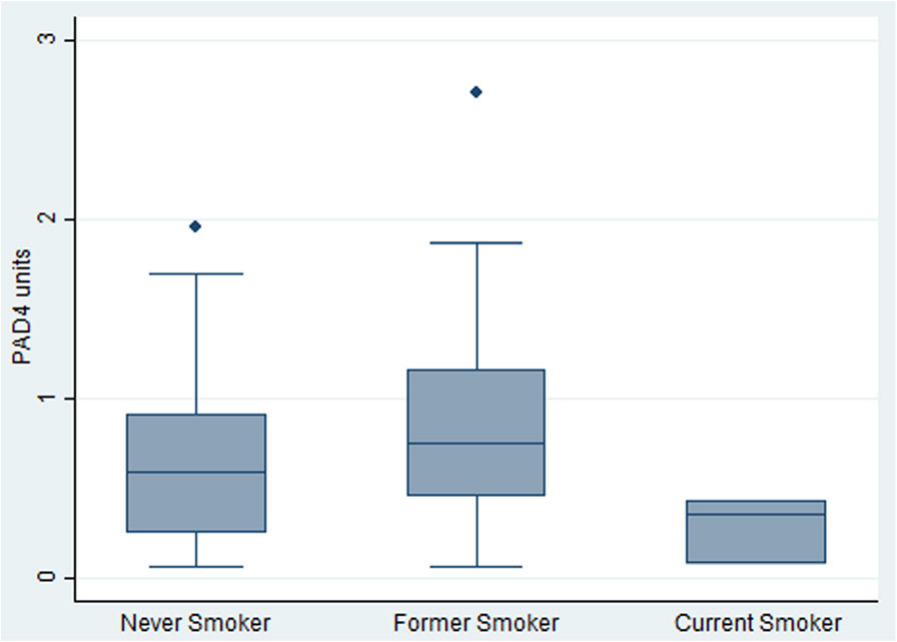
Anti-PAD4 antibody units in patients with positive tests by smoking status. Anti-PAD4 antibody units for the 68 anti-PAD4 positive patients, grouped by smoking history, are shown. The median anti-PAD4 level, interquartile range, and outliers are indicated in box plots for never (*n* = 40), former (*n* = 25), and current smokers (*n* = 3)

Considering the demographic and clinical similarities among people in the anti-PAD4 mono-reactive and anti-PAD3/4 cross-reactive antibody positive subgroups, these patients were combined into a single “anti-PAD4 positive” group for subsequent analyses exploring the link between having *any* anti-PAD4 antibodies and smoking. To further define the relationship between smoking and anti-PAD4 antibodies, the level of PAD4 antibodies in those individuals who were anti-PAD4 seropositive was evaluated according to smoking history (Fig. 1). Current smokers had a median anti-PAD4 antibody level of 0.35 units (IQR 0.07–0.42), former smokers had a median of 0.75 units (IQR 0.45–1.16), and the median level in never smokers was 0.58 (IQR 0.24–0.91) (Fig. 1). Though median anti-PAD4 units were lowest in current smokers, differences between the groups were not statistically significant (*p* = 0.14).

Univariate associations between clinical variables and PAD4 antibody status were evaluated between patients with and without anti-PAD4 autoantibodies (Table 2) and revealed no significant associations between smoking history and PAD4 antibodies in unadjusted analyses. In contrast, patients who possessed one or more SE allele had a 2.4-fold increase in odds of having PAD4 antibodies, and those with RA disease duration greater than 10 years were more likely to have PAD4 antibodies by a factor of 3.6 (*p* <  0.01 for both). In addition, patients who were anti-CCP positive were 5.2-times more likely to have anti-PAD4 antibodies (p <  0.01). In a multivariable model including potential risk factors for PAD4 antibody development, that is, sex, race, smoking status, SE status and disease duration, the presence of SE and disease duration greater than 10 years both remained significantly associated with PAD4 antibodies. Smoking history, however, remained unassociated with PAD4 antibodies in the multivariable model (Table 2).

**Table 2:** Associations between demographic and clinical variables and presence of anti-PAD4 antibodies

	Univariate Models		Multivariable Model*	
Variable	OR (95% CI)	*p*-value	OR (95% CI)	*p*-value
Female sex	1.1 (0.6–2.2)	0.74	0.9 (0.4–1.9)	0.74
Non-Caucasian Race	1.0 (0.5–1.9)	0.87	1.5 (0.7–3.2)	0.34
Ever Smoker	0.8 (0.5–1.4)	0.46	0.8 (0.4–1.4)	0.41
Disease duration > 10 years	3.6 (2.0–6.5)	< 0.01	3.5 (1.9–6.3)	< 0.01
Presence of Shared epitope	2.4 (1.3–4.3)	< 0.01	2.3 (1.2–4.5)	0.02

*OR* odds ratio, *CI* confidence interval; *model adjusted for disease duration, SE, race, smoking status, and sex

Due to the strong association of anti-PAD4 antibodies with SE and disease duration in univariate and multivariate analyses (Table 2), stratified analyses were performed to examine the influence of smoking history on these relationships (Table 3). In subgroups with a disease duration less than or greater than 10 years, there was no significant association between smoking history and presence of PAD4 antibodies. Even among patients with early RA (i.e. disease duration less than two years), where a history of smoking may be temporally closer to antibody development, smoking remained unassociated with PAD4 antibodies (*n* = 34, OR = 0.20 [0.02–2.05], *p* = 0.18). In those with one or more SE alleles, smoking history was associated with a decreased odds of having PAD4 antibodies, but this was not statistically significant (OR = 0.7 [0.3–1.3]; *p* = 0.25).

**Table 3:** Associations between anti-PAD4 antibody presence and ever smoking in stratified analyses

Variable	OR (95% CI)	*p*-value
With Shared Epitope	0.7 (0.3–1.3)	0.25
No Shared Epitope	0.9 (0.3–2.6)	0.90
Duration > 10	0.9 (0.4–1.9)	0.75
Duration < 10	0.8 (0.3–2.0)	0.66

*OR* Odds ratio from univariate logistic regression, *CI* confidence interval

To understand if smoking history or disease duration influenced the development of anti-PAD4 antibodies in patients with SE alleles, these relationships were further examined in stratified analyses (Table 4). Interestingly, there was a significant association between having SE alleles and developing PAD4 antibodies in never smokers (OR = 2.7 [1.2–6.0]; *p* = 0.01) (Table 4), but not in individuals who had a history of smoking. A similar risk of developing anti-PAD4 antibodies among SE-positive individuals was also observed in those with a disease duration greater than 10 years (OR = 2.7 [1.1–6.4]; *p* = 0.03).

**Table 4:** Associations between anti-PAD4 antibody presence and shared epitope in stratified analyses

Variable	OR (95% CI)	*p*-value
In Ever Smokers	2.0 (0.8–5.1)	0.16
In Never Smokers	2.7 (1.2–6.0)	0.01
Duration > 10	2.7 (1.1–6.4)	0.03
Duration < 10	1.5 (0.6–3.6)	0.40

*OR* Odds ratio from univariate logistic regression, *CI* confidence interval

## Discussion

In this cross-sectional study of patients with RA, smoking history was not associated with having anti-PAD4 antibodies or the anti-PAD3/4 cross-reactive antibody subset. This lack of association persisted when controlling for potentially confounding factors in multivariable and stratified analyses. These findings were contrary to our hypothesis and suggested a possible inverse relationship between smoking and the development of anti-PAD4 antibodies. This is supported by the findings that there were no current smokers among patients with anti-PAD3/4 antibodies and that current smokers had the lowest levels of anti-PAD4 antibodies. In addition, there was a significant association between SE and anti-PAD4 antibody development only in never smokers, but not in those with a history of smoking. This study also confirmed previously observed associations of anti-PAD4 and anti-PAD3/4 antibodies with longer disease duration and anti-CCP antibodies [19, 20, 23, 26]. Taken together, these results suggest that environmental factors, other than cigarette smoking, may drive the development of anti-PAD4 antibodies in patients with RA.

This is the first study to systematically evaluate the relationship between smoking and the development of anti-PAD4 and anti-PAD3/4 antibodies in patients with RA. Although not previously appreciated, detailed review of clinical data from several published RA studies suggests a persistently lower smoking history among patients with anti-PAD4 or anti-PAD3/4 antibodies. Borderline and significant inverse associations of anti-PAD4 antibodies with a history of ever smoking were observed in indigenous North American (*p* = 0.067) [20] and primarily Caucasian (*p* = 0.016) [23] RA cohorts, respectively. Furthermore, in the two published studies of anti-PAD3/4 antibodies, current smokers were less prevalent in the anti-PAD3/4 antibody positive group compared to those without anti-PAD antibodies (0% vs. 16%; *p* = 0.075 and 15% vs. 33%; *p* = 0.13) [24, 25]. These data bolster the findings of our current study and strongly suggest that smoking is not the trigger of anti-PAD4 antibody development in patients with RA.

These findings are surprising given the strong association of anti-PAD4 antibodies and anti-CCP observed in this and published studies, as well as the widely accepted relationship between smoking and anti-CCP development [19, 20, 23, 25–27]. However, although initial studies suggested an association of smoking history with ACPA development, more recent studies focusing on isotypes of anti-CCP antibodies and specific ACPAs have painted a more complex picture [28]. For example, in one study of patients with RA, smoking was not associated with anti-CCP, citrullinated vimentin or citrullinated fibrinogen antibodies, but was associated with citrullinated alpha-enolase peptide antibodies [29]. Another recent study demonstrated that only IgG2 and IgA isotypes of anti-CCP antibodies associated with smoking, while IgG1 and IgG4 anti-CCP antibodies were associated with having SE [30]. These findings suggest that the relationship between smoking, citrullination, and the autoantibody response in RA is more nuanced than previously appreciated.

Understanding how specific environmental stimuli affect the nature of the ACPA response and subsequent development of anti-PAD4 antibodies is important to define environmental factors that drive disease propagation and amplification. The association of anti-PAD4 antibodies with disease duration suggests the presence of persistent drivers of inflammation that may contribute to the evolution of the autoantibody response and subsequent generation of PAD4-activating autoantibodies. These data also may inform the interpretation of the previously observed increased risk of RA-associated ILD in patients with anti-PAD3/4 antibodies and a history of smoking [24]. Rather than smoking being the causal factor for the development of anti-PAD3/4 antibodies and subsequent ILD, one interpretation of the present findings is that of a two-hit model. In this scenario, two independently occurring factors (i.e. smoking and anti-PAD3/4 antibodies) synergize to induce ILD. This model is speculative and requires further studies to: 1) validate the link between anti-PAD3/4 antibodies and ILD; and 2) determine if these antibodies can directly perpetuate lung damage in patients with RA.

These results implicate environmental factors other than smoking in anti-PAD4 antibody development, perhaps through important interactions with SE alleles. In this regard, anti-*Porphyromonas gingivalis* (*Pg*) antibodies were associated with non-smoking status in a recent French cohort of patients with early RA [31], and ACPAs were shown to be associated with SE alleles only in patients who had a previous exposure to *Aggregatibacter actinomycetemcomitans (Aa)* [32], a bacterium linked to periodontitis*.* This work also demonstrated that leukotoxin A, a membranolytic toxin expressed by *Aa*, induces potent activation of the PAD enzymes resulting in hypercitrullination of known RA autoantigens, thus linking periodontal infection to ACPA development [32]. Given the association of both anti-PAD4 antibodies and anti-*Pg* antibodies with non-smoking status in patients with RA, and association of *Aa* with PAD enzyme activation, periodontal disease and resulting immune responses could play important roles in the development of anti-PAD4 antibodies. Exploring this mechanistic link is a key area for future research.

There are important outstanding questions that were not addressed in the current study including the interaction between smoking and anti-PAD4 antibodies in patients with early RA and their relationship to ILD development. Patients in this cohort had long-standing RA that was generally well-controlled with treatment, so those with early RA or poorly-controlled disease were underrepresented, and clinical information regarding the presence of ILD was not available. In addition, several subanalyses were performed as a mechanism to control for potential confounders and ensure that an association of anti-PAD4 antibodies with smoking in important patient subgroups was not overlooked. While this strengthened the conclusion that smoking is not linked to anti-PAD4 development, multiple subanalyses have the caveat of reducing the sample size and thus the power to determine positive associations. Future studies are needed to determine if these results are applicable to other cohorts including early RA patients and those with ILD. Lastly, this study was cross-sectional with anti-PAD4 antibody testing performed at a single point in time, so could not capture future anti-PAD4 antibody development in these individuals. Since these antibodies are associated with disease duration, a follow-up study measuring seroconversion to anti-PAD4 antibody positivity over time in a longitudinal cohort may reveal novel information regarding the relationship between environmental factors and anti-PAD4 antibody development.

## Conclusions

This study demonstrated that anti-CCP, SE alleles, and disease duration, but not a history of smoking, were associated with anti-PAD4 antibodies in patients with RA. Further efforts aimed at defining the inciting environmental stimuli linked to the development of anti-PAD4 in RA patients are warranted and may reveal important insights into disease mechanism. Defining the environmental factors associated with the generation of anti-PAD4 and anti-PAD3/4 antibodies may also identify novel targets for therapeutic intervention to prevent the generation of these potentially disease-amplifying autoantibodies in patients with RA.

## Abbreviations

Aa: Aggregatibacter actinomycetemcomitans
ACPA: anti-citrullinated protein antibody
ANOVA: analysis of variance
CCP: cyclic citrullinated peptide
CDAI: clinical disease activity index
DNA: deoxyribonucleic acid
ELISA: enzyme-linked immunosorbent assay
HLA: human leukocyte antigen
ILD: interstitial lung disease
PAD: peptidylarginine deiminase
PCR: polymerase chain reaction
Pg: Porphyromonas gingivalis
RA: rheumatoid arthritis
RF: rheumatoid factor
SE: shared epitope
VAS: visual analog scale
